# Metformin ameliorates scleroderma via inhibiting Th17 cells and reducing mTOR-STAT3 signaling in skin fibroblasts

**DOI:** 10.1186/s12967-021-02860-z

**Published:** 2021-05-04

**Authors:** Jeonghyeon Moon, Seon-yeong Lee, Jeong Won Choi, A Ram Lee, Jin Hee Yoo, Su-Jin Moon, Sung-Hwan Park, Mi-La Cho

**Affiliations:** 1 Lab of Translational ImmunoMedicine, Catholic Research Institute of Medical Science, College of Medicine, The Catholic University of Korea, Seoul, 06591 Republic of Korea; 2 Rheumatism Research Center, Catholic Research Institute of Medical Science, College of Medicine, The Catholic University of Korea, 222 Banpo-Daero, Seocho-gu, Seoul, 06591 Republic of Korea; 3 Department of Biomedicine & Health Sciences, College of Medicine, The Catholic University of Korea, 222, Banpo-daero, Seocho-gu, Seoul, 06591 Republic of Korea; 4 Divison of Rheumatology, Department of Internal Medicine, Uijeongbu St. Mary’s Hospital, College of Medicine, The Catholic University of Korea, Uijeongbu, 11765 Republic of Korea; 5 Division of Rheumatology, Department of Internal Medicine, Seoul St. Mary’s Hospital, College of Medicine, The Catholic University of Korea, Seoul, 06591 Republic of Korea; 6 Department of Medical Lifescience, College of Medicine, The Catholic University of Korea, 222, Banpo-daero, Seocho-gu, Seoul, 06591 Republic of Korea

**Keywords:** Metformin, Scleroderma, Inflammation, STAT3, MTOR

## Abstract

Scleroderma is an autoimmune disease that causes dermal fibrosis. It occurs when collagen accumulates in tissue as a result of persistent inflammation. Th17 cells and pro-inflammatory cytokines such as IL-1β, IL-6, IL-17, and TNF-α play important roles in the pathogenesis of scleroderma. Because metformin, a medication used to treat diabetes, has effective immunoregulatory functions, we investigated its therapeutic function in scleroderma. Mice in a model of bleomycin-induced scleroderma were treated with metformin for 2 weeks. Histological assessment demonstrated protective effects of metformin against scleroderma. Metformin decreased the expression of pro-inflammatory factors in dermal tissue and lymphocytes. It also decreased mRNA expression of pro-inflammatory cytokines (IL-1β, IL-6, IL-17, and TNF-α) and fibrosis-inducing molecules both in vivo and in vitro. These results suggest that metformin treatment has anti-inflammatory effects on lymphocytes via the inhibition of IL-17 and cytokines related to Th17 differentiation, such as IL-1β, IL-6, and TNF-α. To investigate how metformin modulates the inflammatory process in skin fibroblasts, we measured mTOR-STAT3 signaling in skin fibroblasts and found that phosphorylated mTOR and phosphorylated STAT3 protein expression were decreased by metformin treatment. These results suggest that metformin has potential to treat scleroderma by inhibiting pro-inflammatory cytokines and anti-inflammatory activity mediated by mTOR-STAT3 signaling.

## Introduction

Scleroderma is an autoimmune disease that causes thickening of the skin, stiffness, exhaustion, and abnormal blood flow [1–3]. Although the pathogenesis of scleroderma remains unclear, pro-inflammatory cytokines, the tissue microenvironment, and inflammation are contributing factors [4, 5].

T cells seem to be closely associated with the pathogenesis of scleroderma [6]. Collagen, the main effector molecule in the synthesis of sclerosis, is more abundant in areas of mononuclear cell infiltration [7]. Soluble factors secreted by inflammatory cells may enhance the deposition of collagen [8]. In particular, IL-17 and Th17 cells, which are involved in autoimmune diseases such as rheumatoid arthritis [9], psoriasis [10] and lupus erythematosus [11], also play a critical role in scleroderma [12–14]. A previous study showed that the secretion of IL-17A contributed to immune inflammation and the pathogenesis of scleroderma in an animal model [15]. Therefore, therapies that focus on inhibiting Th17 cells may be promising for treating scleroderma.

Th17 cells produce pro-inflammatory cytokines such as IL-17A, IL-17F, IL-21, and IL-22 [16]. Their differentiation and maturation are facilitated by a combination of various cytokines and the microenvironment [17]. Combinations of transforming growth factor (TGF)-β and IL-6; IL-21 and TGF-β; or IL-1, IL-6, and IL-23 have all been proposed as cytokine mixes that induce helper T cell differentiation toward the Th17 subset by manipulating signal transducer and activator of transcription (STAT3) signaling and expression of the master transcription factors RAR-related orphan receptor gamma T (RorγT) and aryl hydrocarbon receptor (AHR) [18–20].

Metformin, which was first introduced as a biguanide antidiabetic medication [21]], is widely used to treat diabetic patients [22]. It is interesting that its anti-inflammatory effects are due to its activation of AMP-activated protein kinase (AMPK), a major sensor that modulates lipid and glucose metabolism [23, 24]. Metformin has the effect of modulating the immune response by activating AMPK by phosphorylation, while it regulates autophagy, epigenetic regulation, protein synthesis, and cell survival through the NF-κB/mTOR signaling as an AMPK-independent pathway [25]. Activated AMPK by metformin inhibits mTOR expression. It leads to inhibition of STAT3. In consequence, it decreases Th17 generation, germinal center formation and monocyte to macrophage differentiation, which is regulated by STAT3. On the other hand, Treg generation and macrophage M2 polarization are increased [26, 27].

Our previous studies demonstrated that metformin suppresses the development of lung fibrosis and inflammatory bowel disease by regulating the Th17–IL-17 axis [28]. Metformin regulates the T cell differentiation in vitro and in vivo [29, 30]. AMPK activated by metformin suppresses mammalian target of rapamycin (mTOR), a serine/threonine protein kinase that helps regulate Th17 cells [31, 32]. These findings suggest that metformin has therapeutic potential in scleroderma.

To identify the therapeutic effects of metformin on scleroderma, we used a bleomycin (BLM)-induced murine model of scleroderma. Metformin ameliorates scleroderma in similar mouse models; however, the detailed mechanisms remain unclear [33, 34]. In this study, we demonstrated that metformin ameliorates scleroderma in a mouse model by inhibiting Th17 cells via the regulation of mTOR-STAT3 signaling.

## Materials and methods

### Animals

Female BALB/c mice 8 to 10 weeks old were used in this experiment. All mice were purchased from OrientBio (Sungnam, Korea). They were fed standard mouse chow (Ralston Purina, St. Louis, MO, USA) and water ad libitum. All experimental procedures were reviewed and approved by the Animal Research Ethics Committee of the Catholic University of Korea (Seoul, Korea).

### BLM and metformin treatment

The mice were anesthetized with isoflurane, and then their backs were shaved. They were given daily subcutaneous injections of BLM for 4 weeks (Dong-A Pharm, Seoul, Korea). BLM was sterilized by filtration before injection under the shaved back skin at a dose of 1 mg/mL in PBS. Metformin treatment commenced 2 weeks after the first injection of BLM. 100 mg/kg metformin was injected by oral gavage to mice (*n* = 5 or 6 per group) every day for 2 weeks. Then mice were sacrificed the day after the final treatment. Back skin and lung tissue were acquired and fixed in 10% formalin solution for histological analysis. Spleen tissue was preserved for cryosection.

### Histological assessment

Harvested tissue was fixed in 10% formalin and embedded in paraffin. Sections (6 µm thick) were stained with H&E and Masson’s trichrome (MT). MT staining was conducted using ready–to-use kit (Trichrome Stain (Masson) Kit, HT15, Sigma-Aldrich). Briefly, the tissue was sliced and placed on standard microscopy slides. After deparaffinisation and rehydration, the slides were immersed in Bouin’s solution (HT 10132, Sigma-Aldrich) at 56 °C for 15 min. Subsequently, the slides were washed with tap water for 5 min. Next, the tissues were stained in Weigert’s hematoxylin for 5 min, and then washed again with tap water for 5 min. Then, the slides were stained in Biebrich scarlet-acid fuchsin for 5 min, rinsed in distilled water, incubated in phosphotungstic-phosphomolybdic acid for 5 min, dyed with aniline blue for 5 min, and fixed in 1% acetic acid for 2 min. Finally, the slides were washed in distilled water, dehydrated and mounted. Dermal thickness was measured as described previously [35]. Lung sections were analyzed, and the severity of fibrosis was scored as described previously [36]. For the quantification of collagen, mouse skin and lung tissue were hydrolyzed and analyzed with hydroxyproline assays.

### Immunohistochemistry

Immunohistochemistry was performed with VECTASTAIN ABC kits (Vector Laboratories, Burlingame, CA, USA). Tissue was incubated with primary antibodies against IL-6, IL-17, TGF-β, STAT3, phosphorylated mTOR (p-mTOR), phosphorylated AMPK (p-AMPK), and α-smooth muscle actin (α-SMA) overnight at 4 °C. The primary antibody was detected with a biotinylated secondary linking antibody, followed by incubation with a streptavidin-peroxidase complex for 1 h. The final color was produced with DAB chromogen (Dako, Carpinteria, CA, USA). Positive cells, which were identified by a dark brown deposit in the nucleus, were enumerated in 10 randomly selected high-power fields (HPFs; 400×; 2.37 mm^2^). The sections were counterstained with hematoxylin and photographed with an Olympus photomicroscope (Tokyo, Japan).

### Confocal microscopy

Spleen tissue sections 7 µm in thickness were used for immunostaining. To analyze populations of Th17 cells, we used fluorescein isothiocyanate (FITC)-conjugated anti-CD4 and PE-conjugated anti-IL-17 antibodies (eBioscience, San Diego, CA, USA). Stained sections were observed with a Zeiss microscope (LSM 510 Meta; Carl Zeiss, Oberkochen, Germany) at × 400 magnification. Positive cells were counted, and values were expressed as means ± standard deviations.

### Real-time PCR

mRNA was extracted with TRI Reagent (Molecular Research Center, Cincinnati, OH, USA) according to the manufacturer’s instructions. Complementary DNA was synthesized with a SuperScript Reverse Transcription System (Takara Bio, Kyoto, Japan). A Light-Cycler 2.0 (software version 4.0; Roche Diagnostics, Mannheim, Germany) was used for PCR amplification. All reactions were performed with LightCycler FastStart DNA Master SYBR Green I Mix (Takara) following the manufacturer’s instructions. The following primers were used to amplify mouse genes: for IL-1β, 5′-GGA TGA GGA CAT GAG CAC ATT C-3′ (sense) and 5′-GGA AGA CAG GCT TGT GCT CTG A-3′ (antisense); for IL-6, 5′-ATG CTC CCT GAA TGA TCA CC-3′ (sense) and 5′-TTC TTT GCA AAC AGC ACA GC-3′ (antisense); for IL-17, 5′-CCT-CAA-AGC-TCA-GCG-TGT-CC-3′ (sense) and 5′-GAG-CTC-ACT-TTT-GCG-CCA-AG-3′ (antisense); for TNF-α, 5′-ATG AGC ACA GAA AGC ATG ATC-3′ (sense) and 5′-TAC AGG CTT GTC ACT CGA ATT-3′ (antisense); for TGF-β, 5′-GCC TGA GTG GCT GTC TTT TGA-3′ (sense) and 5′-CAC AAG AGC AGT GAG CGC TGA A-3′ (antisense); for Col1a, 5′-ATG GGA GGA GAG CGT GTG-3′ (sense) and 5′-GAG GTC GGA GAG CAG AGG-3′ (antisense); for β-actin, 5′-GTA CGA CCA GAG GCA TAC AGG-3′ (sense) and 5′-GAT GAC GAT ATC GCT GCG CTG-3′ (antisense). All mRNA levels were normalized to that of β-actin.

### Murine splenocytes isolation and stimulation

Splenocytes were acquired from spleen tissue of BALB/c mice and sieved through a mesh screen. Then red blood cells were lysed in hypotonic ACK buffer. The remaining splenocytes were maintained in RPMI 1640 medium containing 5% fetal bovine serum. Splenocytes were stimulated with plate-bound anti-CD3 (0.5 µg/mL; BD Pharmingen) for 3 days. Metformin was obtained from Sigma-Aldrich (St. Louis, MO, USA) and dissolved in RPMI 1640 medium including 5% fetal bovine serum. Metformin was administered during the same 3-day period as the anti-CD3 stimulation.

### Western blotting

Skin fibroblasts from a healthy donor were cultured with 10 ng/ml of IL-6 and metformin for 1 h. Proteins were isolated by sodium dodecyl sulfate–polyacrylamide gel electrophoresis and transferred onto nitrocellulose membranes (Amersham Pharmacia Biotech, Piscataway, NJ, USA). Western blotting was performed with a SNAP i.d. Protein Detection System (Millipore, Billerica, MA, USA). Membranes were stained with primary antibodies against phosphorylated mTOR (p-mTOR; Ser2448) (D9C2), phosphorylated STAT3 (p-STAT3; 727), and β-actin (all from Cell Signaling Technology, Danvers, MA, USA).

### Statistical analysis

Data are presented as means ± SDs of at least three independent experiments or at least three independent samples, with five mice in each group. In vitro experiments were independently repeated three or more times, and each experiment had at least three samples. One-way analysis of variance followed by Bonferroni’s post hoc test was used to compare differences among three or more groups. The Mann–Whitney U test was used to compare numerical data between groups. To assess the Gaussian distribution and equality of variance, we used the Shapiro–Wilk test and Levene’s test, respectively. P < 0.05 was considered statistically significant. Statistical analyses were performed with IBM SPSS Statistics 20 for Windows (IBM, Armonk, NY, USA).

## Results

### Metformin alleviates scleroderma in a BLM-induced mouse model

**Fig. 1:**
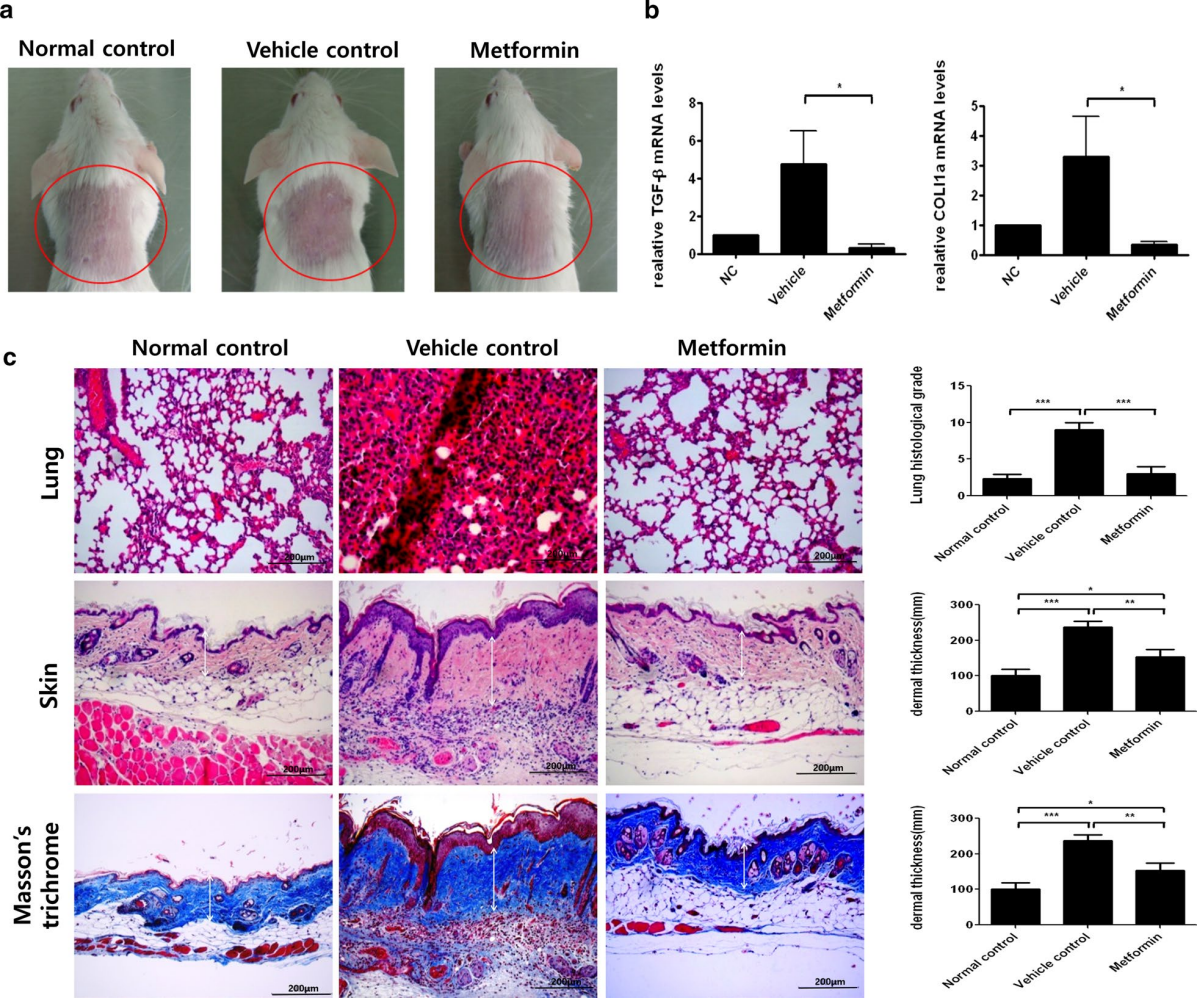
Metformin treatment decreases the stiffness of tissue in BLM-induced mice. **a** The back-skin morphology of mice with BLM-induced scleroderma in each group is shown. **b** mRNA expression of fibrosis markers in skin tissue was measured. **c** H&E staining and MT images reveal the therapeutic effects of metformin in mouse skin and lung tissue. White arrows indicate skin thickness (H&E) and cutaneous fibrosis (MT). *P < 0.05, **P < 0.01, and ***P < 0.005. Scale bars = 200 μm

To assess whether metformin has therapeutic potential in scleroderma, we injected BALB/c mice with BLM for 4 weeks. Metformin treatment commenced 2 weeks after the first BLM injection. Metformin-treated groups had less skin damage and more hair growth than vehicle groups (Fig. 1a). We measured TGF-β and Col1a, markers of skin tissue fibrosis, with quantitative PCR. The results showed that metformin significantly decreased TGF-β and Col1a mRNA expression (Fig. 1b). H&E staining of lung and skin tissue revealed that metformin reduced lung fibrosis and skin thickness, findings we confirmed with MT analyses (Fig. 1c). These results show that metformin counteracts the pathogenesis of scleroderma.

### Metformin reduces the expression of pro-inflammatory cytokines in a mouse model of scleroderma

**Fig. 2:**
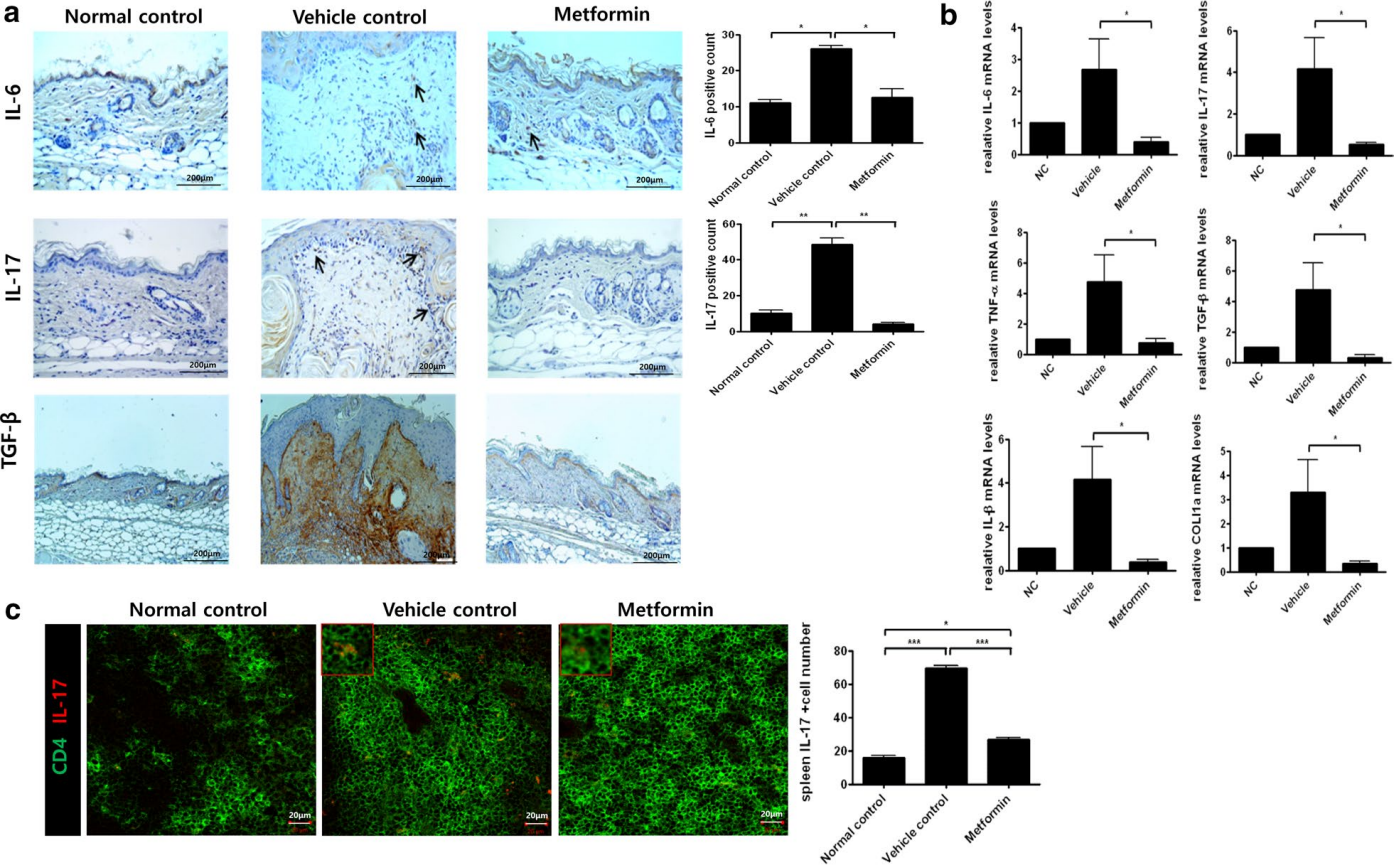
Metformin regulates pro-inflammatory cytokines in mice with scleroderma. **a** Immunohistochemistry was used to detect the presence of infiltrating lymphocytes and proinflammatory cytokines, such as IL-6, IL-17, and TGF-β, in skin tissue. Scale bars = 200 μm. **b** Splenocyte proinflammatory cytokines were detected by quantitative PCR. **c** Spleen tissue was stained with anti-CD4 and anti-IL-17 antibodies to identify Th17 cells. Arrows indicate the stained cells. *P < 0.05, **P < 0.01, and ***P < 0.005. Scale bars = 20 μm

To investigate the effects of scleroderma on immune cells, we used immunohistochemistry to measure the infiltration of lymphocytes into skin tissue. Metformin treatment reduced not only the infiltration of lymphocytes but also the expression of pro-inflammatory cytokines (Fig. 2a). Furthermore, mRNA expression of IL-1β, IL-6, IL-17, TGF-β, TNF-α, and Col1a in the skin tissues decreased under metformin treatment (Fig. 2b). Immunofluorescence analyses showed that metformin decreased the abundance of Th17 cells in spleen tissue (Fig. 2c). These results demonstrate the regulatory effects of metformin on immune cells.

### Metformin regulates STAT3/mTOR/AMPK signaling in a mouse model of scleroderma

**Fig. 3:**
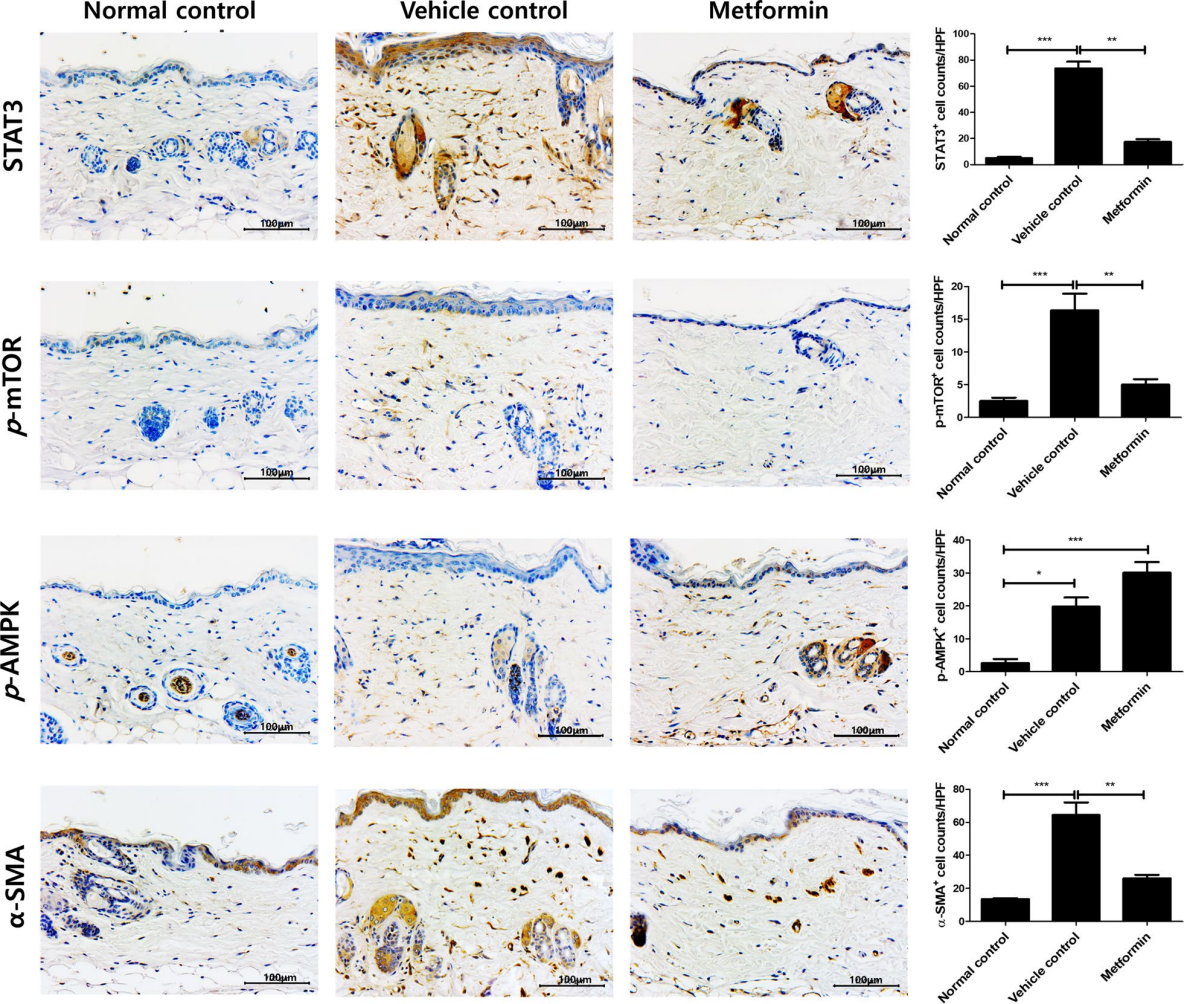
Metformin regulates mTOR-STAT3 signaling in skin fibroblasts. Immunohistochemistry was used to characterize the expression of STAT3, p-mTOR, p-AMPK, and α-SMA in skin tissue in BLM-induced mice. Arrows indicate the stained cells. *P < 0.05, **P < 0.01, and ***P < 0.005. Scale bars = 100 μm

Samples of skin tissue were analyzed by immunohistochemistry to determine the effects of metformin on the skin of BLM-induced mice. The results showed that metformin significantly reduced the expression of STAT3 and p-mTOR in skin. However, the expression of p-AMPK tended to increase under metformin treatment. The expression of α-SMA, a fibrosis marker, was significantly attenuated in metformin-treated mice (Fig. 3). These data suggest that metformin inhibits the pathogenesis of scleroderma by modulating mTOR-STAT3 signaling.

### The expression of pro-inflammatory cytokines of splenocytes is decreased under in vitro anti-CD3 conditions

**Fig. 4:**
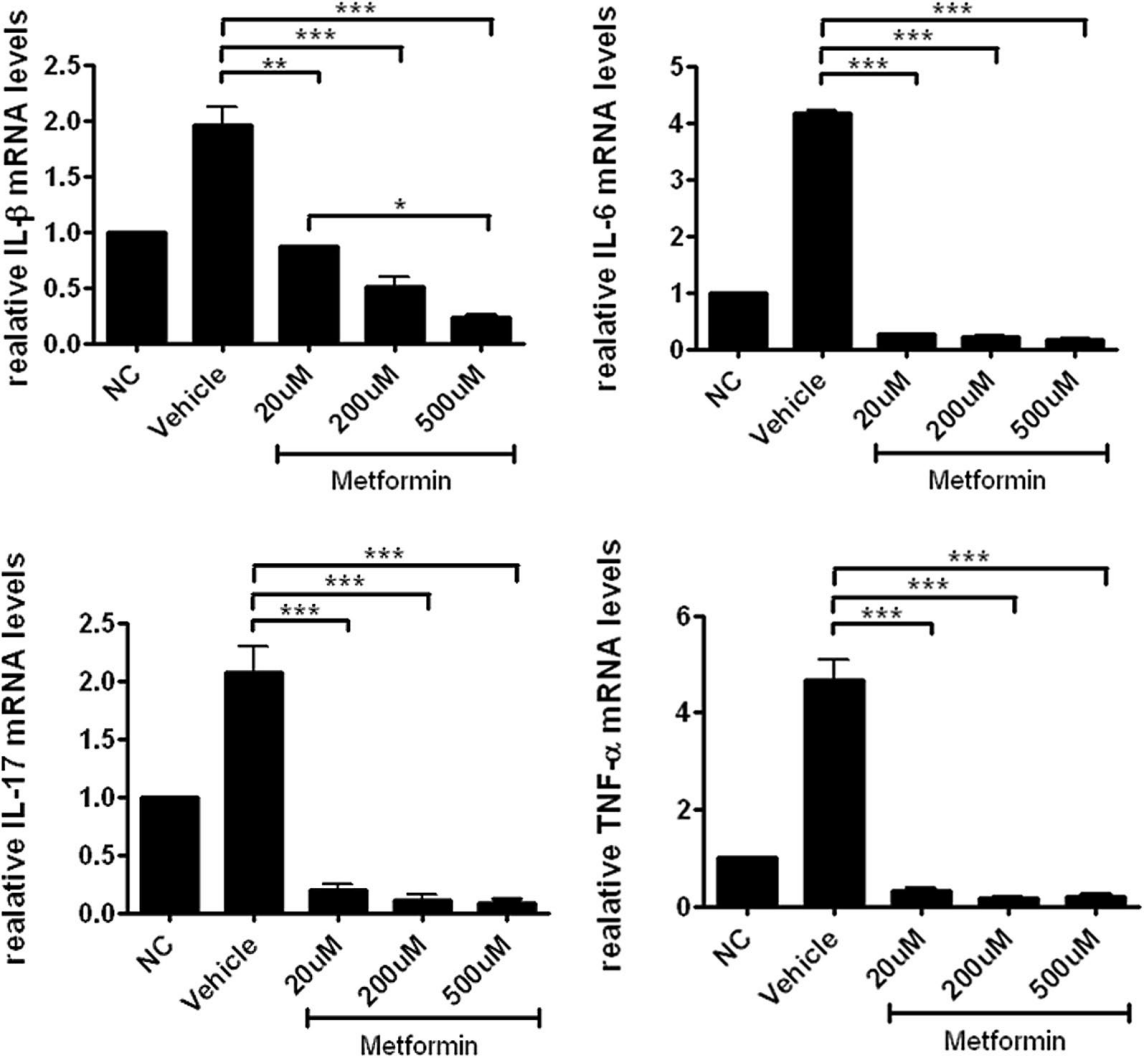
Metformin inhibits mRNA expression of Th17 differentiation-related genes in murine splenocytes. Levels of IL-17, IL-1β, IL-6, and TNF-α in stimulated cells were reduced in metformin-treated groups under anti-CD3 stimulation. *P < 0.05, *P < 0.05, **P < 0.01, and ***P < 0.005

To determine whether metformin inhibits Th17 differentiation, we stimulated murine splenocytes with anti-CD3. Three days later, mRNA expression of stimulated cells was detected with quantitative PCR. The results showed that metformin suppressed levels of the pro-inflammatory cytokines IL-1β, IL-6, IL-17, and TNF-α (Fig. 4), which indicates that it has inhibitory effects on T cells.

### Metformin inhibits activation of mTOR-STAT3 signaling in skin fibroblasts

**Fig. 5:**
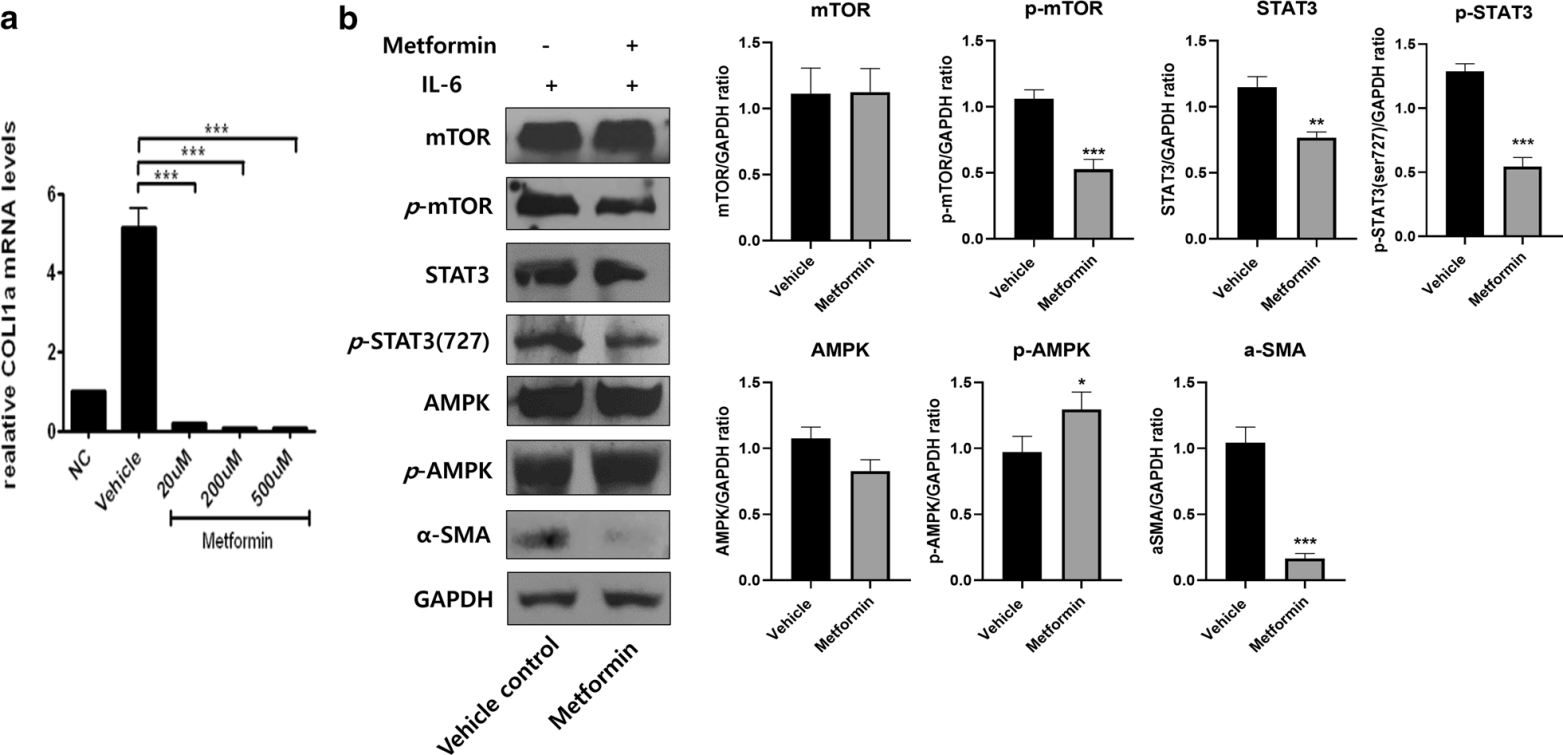
Metformin regulates the expression of Col1a, p-STAT3, and p-mTOR in skin fibroblasts. **a** The expression of Col1a, a fibrosis marker, was inhibited by metformin treatment. **b** The protein levels of mTOR, p-mTOR, STAT3, p-STAT3, AMPK, p-AMPK, α-SMA and GAPDH in skin fibroblasts is presented by western blotting in the presence of IL-6. *P < 0.05, **P < 0.01, and ***P < 0.005

To investigate the mechanism through which metformin has anti-fibrosis and anti-inflammatory function, metformin was treated in STAT3 activated-fibroblasts by IL-6 treatment. Our previous studies showed that IL-6 provides fibroblasts with an inflammatory condition in in vitro experiment (data not shown). The expression of Col1a, a fibrosis-inducing molecule, was also decreased in metformin-treated skin fibroblasts (Fig. 5a). To further investigate the mechanism behind the regulatory effects of metformin on skin, we used Western blotting to investigate upstream signaling pathways for inflammatory cytokine production in skin fibroblasts (Fig. 5b). The protein levels of mTOR, p-mTOR, STAT3, p-STAT3, AMPK and p-AMPK were detected. We found the decreased protein levels of both p-STAT3 and p-mTOR and increase of p-AMPK under metformin treatment. Besides, α-SMA which is the fibrosis marker is decreased by the metformin treatment. These results show that metformin has protective effects against scleroderma via the regulation of mTOR-STAT3 signaling.

## Discussion

Scleroderma is a systemic autoimmune disease that damages the skin, lung, and other tissue through chronic inflammation [6]. Th17 cells are the main cause of immunological disorders, including scleroderma [37]. In previous studies, metformin exhibited therapeutic effects for autoimmune diseases [38, 39]. The activated T cells and circulatory Th17 cells were increased in systemic sclerosis patients compared to healthy control [40]. Although the scleroderma development mechanism of the rodent model has notable differences between human mechanisms and in vitro experiments, the bleomycin-induced mouse model is widely used [35]. Our previous studies have shown that metformin has preventive effects on autoimmune disorders such as rheumatoid arthritis [41], Sjogren syndrome [42], inflammatory bowel disease [43] and lupus erythematosus [44]. Hence, we investigated the potential anti-fibrosis and immunoregulatory effects of metformin on scleroderma.

In previous studies, metformin has an anti-fibrotic effect on skin fibrosis through the decrease of Col1a and α-SMA [45–47]. Hence, we used animal model and treated BLM-induced BALB/c mice with metformin for 2 weeks, beginning 2 weeks after the first BLM injection. Metformin-treated mice had lower TGF-β and Col1a mRNA expression than the vehicle group. The dermal thickness of metformin-treated groups was improved compared to that of untreated control groups. Furthermore, pulmonary fibrosis was reduced in metformin-treated mice.

BLM-induced mice were first designed as an animal model for pulmonary fibrosis [48]. Hence, we used H&E staining to examine lung tissue and found that fibrosis was significantly decreased in metformin-treated mice.

Pro-inflammatory cytokines play a role in the pathogenesis of scleroderma [49, 50]. To identify the immunoregulatory effects of metformin in a mouse model of scleroderma, we analyzed skin tissue by immunohistochemistry. We found that expression of the pro-inflammatory cytokines IL-6, IL-1β, IL-17, and TNF-α decreased in metformin-treated mice. Furthermore, mRNA expression of the fibrosis markers TGF-β and Col1a decreased under metformin treatment. The abundance of Th17 cells in spleen tissue was also diminished by metformin.

To elucidate the mechanistic basis for the immunoregulatory effects of metformin in scleroderma, we investigated upstream pathways. Although its activity is not completely understood, metformin acts as an AMPK activator [51]. AMPK is a serine/threonine protein kinase that plays a critical role in cellular metabolism, maintenance of the energy balance, inflammation, neurodegenerative disease, and pain [52, 53]. Moreover, it inhibits STAT3 signaling [54]. Activated STAT3 promotes inflammation by increasing pro-inflammatory cytokines [55]. mTOR-STAT3 signaling induces IL-17 expression via interferon regulatory factor 4 (IRF-4) [56, 57]. In addition, α-SMA is known to be a fibrosis marker. Metformin ameliorates fibrosis via a decrease of α-SMA and regulation of oxidative stress [58]. However, it has a limitation that it cannot provide a wider sight of which mechanism works in the previous study. Our immunohistochemical analyses of skin tissue showed that metformin significantly decreased STAT3 and mTOR expression, whereas AMPK expression tended to increase in metformin-treated mice skin tissue, likely leading to decreased α-SMA expression. However, the protein levels of phosphorylated AMPK which is an activated form of AMPK were increased in metformin-treated cells.

To investigate how metformin inhibits Th17 differentiation, we treated splenocytes with metformin under anti-CD3 stimulation for 3 days. Then we detected cellular mRNA expression of IL-1β, IL-6, IL-17, and TNF-α by quantitative PCR. mRNA expression of these pro-inflammatory cytokines was significantly attenuated by metformin at all doses. Metformin also inhibited the production of IL-17, which is the main product of Th17 cells. These data suggest that metformin has anti-inflammatory functions in immune cells.

IL-6 phosphorylates STAT3 by activating JAK and mTOR. Phosphorylated STAT3 promotes the secretion of pro-inflammatory cytokines and induces inflammation [59]. We found that metformin reduced the expression of Col1a and α-SMA in skin fibroblasts. To determine how metformin inhibits the expression of mTOR and STAT3 in skin fibroblasts under inflammatory conditions, we treated fibroblasts with IL-6 and found that metformin decreased protein levels of p-mTOR and p-STAT3. These results demonstrate that metformin has both immunoregulatory effects and inhibitory functions in skin. Two other groups have shown that metformin ameliorates scleroderma in BLM-induced mice [33, 34] [33, 34]. However, those studies focused on histological pathology, regulatory T cells, and effector T cells. Our data demonstrate that metformin has immunoregulatory functions via the suppression of Th17 cells. Note that our results are the first to show that metformin modulates STAT3-mTOR signaling in fibroblasts. Collectively, our results suggest that metformin may have therapeutic potential in scleroderma.

## Data Availability

All datasets generated for this study are included in the article.
