# Correction to: Metformin ameliorates scleroderma via inhibiting Th17 cells and reducing mTOR-STAT3 signaling in skin fibroblasts

**DOI:** 10.1186/s12967-021-02916-0

**Published:** 2021-06-21

**Authors:** Jeonghyeon Moon, Seon-yeong Lee, Jeong Won Choi, A. Ram Lee, Jin Hee Yoo, Su-Jin Moon, Sung-Hwan Park, Mi-La Cho

**Affiliations:** 1 Lab of Translational ImmunoMedicine, Catholic Research Institute of Medical Science, College of Medicine, The Catholic University of Korea, Seoul, 06591 Republic of Korea; 2 Rheumatism Research Center, College of Medicine, Catholic Research Institute of Medical Science, The Catholic University of Korea, 222 Banpo-Daero, Seocho-gu, Seoul, 06591 Republic of Korea; 3 Department of Biomedicine & Health Sciences, College of Medicine, The Catholic University of Korea, 222, Banpo-daero, Seocho-gu, Seoul, 06591 Republic of Korea; 4 Divison of Rheumatology, Department of Internal Medicine, Uijeongbu St. Mary’s Hospital, College of Medicine, The Catholic University of Korea, Uijeongbu, 11765 Republic of Korea; 5 Division of Rheumatology, Department of Internal Medicine, Seoul St. Mary’s Hospital, College of Medicine, The Catholic University of Korea, Seoul, 06591 Republic of Korea; 6 Department of Medical Lifescience, College of Medicine, The Catholic University of Korea, 222, Banpo-daero, Seocho-gu, Seoul, 06591 Republic of Korea

## Correction to: J Transl Med (2021) 19:192 https://doi.org/10.1186/s12967-021-02860-z

In the original publication [1] there was an incorrect funding section. The incorrect and correct funding information is published in this correction article. The original article has been updated.

**Incorrect funding**

- This research was supported by a grant of the Korea Health Technology R&D Project through the Korea Health Industry Development Institute (KHIDI) funded by the Ministry of Health & Welfare, Republic of Korea (Grant Number HI20C1496), the National Research Foundation of Korea (NRF) grant funded by the Korea government (MSIT) (No. NRF-2018R1A2B6007291) and (No. NRF-2018R1C1B6005889).

**Correct funding**

- This research was supported by a grant of the Korea Health Technology R&D Project through the Korea Health Industry Development Institute (KHIDI) funded by the Ministry of Health & Welfare, Republic of Korea (Grant Number HI20C1496, HI15C3062), the National Research Foundation of Korea (NRF) grant funded by the Korea government (MSIT) (No. NRF-2018R1A2B6007291) and (No. NRF-2018R1C1B6005889).

## References

[1] Moon J, Lee S, Choi JW, Lee AR, Yoo JH, Moon S-J, Park S-H, Cho ML (2021). Metformin ameliorates scleroderma via inhibiting Th17 cells and reducing mTOR-STAT3 signaling in skin fibroblasts. J Transl Med.

